# The Morphosynthesis of Event Portfolios: Connecting Networks and the Community

**DOI:** 10.3389/fspor.2021.785018

**Published:** 2022-01-21

**Authors:** Vassilios Ziakas

**Affiliations:** Independent Scholar, Leisure Insights Consultancy Ltd, Leeds, United Kingdom

**Keywords:** event portfolio, social networks, community capacity-building, quality of life, transdisciplinary theory

## Abstract

Despite the increasing employment of event portfolios by cities and regions to obtain a number of gains, there is a rather slow response from the academic community to fully understand this complex phenomenon and its potential social value. To address this asymmetry furthering the gap between scholarship and practice, the purpose of this article is to theorize the generative mechanisms that shape portfolios as social systems. Based on up-to-date theoretical development, I attempt in this paper to formulate a comprehensive theory of the integrative portfolio nature that interconnects its contextual, policy, operational, and sociocultural dimensions. I do so, by delineating the connective properties of portfolios to assemble different events and enable stakeholders to work toward the accomplishment of common portfolio goals, and by elaborating on the exigencies of portfolios for contributing to the strengthening of the host community's social fabric. This relational analysis operationalizes latest event portfolio elaborations integrated with the perspectives of community capacity-building and quality of life in order to underpin the formulation of a broader framework accounting for the intersection between strategic portfolio planning and the dynamics of stakeholder attitudes, participation in, and community engagement with portfolios. On these grounds, I suggest a new conceptual lens named “morphosynthesis” to explain the multilevel integration processes that shape event portfolios and enable the interlinking of social networks and the community through the array of events. Morphosynthesis constitutes a transdisciplinary perspective that situates the study of event portfolios as a new autotelic subdiscipline across event management, leisure, sport, culture, and tourism. It provides solid but flexible theoretical logics and heuristic means to navigate in the over-fragmented landscape of events and allied industries with the purpose to enhance their compound social value.

## Introduction

Cities and regions worldwide increasingly invest in staging portfolios of events to achieve multiple benefits. There are several examples, inter alia, such as Edinburgh, Gold Coast, Gothenburg, Auckland, Scotland, and Wales (Antchak et al., 2019). Despite the diffusion of the portfolio concept in practice, there is a disproportionately less response from the academic community on studying the complex phenomenon of event portfolio. In fact, only a modest number of portfolio studies have emerged in the last 15 years, since the phenomenon has been identified and established in the literature (Chalip, 2004; Chalip and Costa, 2005; Getz, 2005, 2008, 2012; Ziakas, 2007, 2010, 2013; Ziakas and Costa, 2010a, 2011a,b). Given the complexity of portfolios, due to the interplay of multiple events and stakeholders with city agendas, ever-changing policy and management realms, challenged by the immanent tensions between economic and socio-cultural orientations (Ziakas, 2014a), the apparent lethargy of the academic community does not provide a much needed multidimensional insight to the phenomenon. This decelerates knowledge creation and theory-building that would benefit the sustainable development of event portfolios, amplify their social value, and their multifaceted contribution to urban or regional prosperity (Ziakas, 2019a).

In response, following the theoretical premises set by Ziakas and Getz (2020, 2021), my contention is that the study of event portfolios requires the building of a transdisciplinary lens, one across and beyond existing subjects, synthesizing thus a new autotelic subdiscipline cutting across event management, leisure, sport, culture, and tourism. To that end, I attempt in this paper to formulate a theory of the integrative portfolio nature with a dual purpose: first, to delineate the connective properties that portfolios entail in order to bring together different events and enable the associated range of stakeholders to work toward the accomplishment of common portfolio goals; and second, to elaborate on the exigencies of portfolios to contribute to the solidification of the host community's social fabric. From this perspective, I intend to account for the bidirectional relationship between portfolios and social order: that is, how portfolios are being affected by the existent social networks in the host community that shape their connective properties, and in turn, how the portfolio may affect the community's social order, building local capacities, and increasing support for, as well as, engagement in joint event-themed efforts for community and tourism development. Thus, I argue that this relational analysis is fundamental for advancing theory and praxis in managing event portfolios as multipurpose policy tools (Ziakas and Costa, 2011b). Relationality is critical because the essence of portfolio management is an integration exercise of the underlying contextual, policy, operational, and sociocultural dimensions (Ziakas, 2014a).

Commencing from the recently established theoretical origins of the event portfolio phenomenon as a multipurpose developmental tool, my analysis moves forward to operationalize the notions of community capacity-building and quality of life merged with the roots of a morphogenetic perspective (Archer, 1995). This scrutiny is substantiated by an exhaustive review of empirical and theoretical work to date on event portfolios. In this regard, I present a conceptual groundwork on the intersection between the factors that affect strategic portfolio planning and the dynamics that shape stakeholder attitudes, participation in, and community engagement with portfolios. The conceptual analysis comprises theoretical elaborations regarding integrative strategic portfolio planning (Ziakas, 2014b), event portfolio network and community capacity-building (Ziakas, 2019b), and portfolio tourism leveraging (Ziakas, 2020) to underpin the building of a broader framework for shaping event portfolios as social systems. In addition, I discuss how the event-related undercurrents of stakeholder attitudes, participation in, and community engagement with events are intertwined with capacity-building. On these grounds, I suggest a new conceptual lens named “morphosynthesis” to explain the multilevel integration processes that shape event portfolios. Through this prism, the interactions of connective properties are viewed as a whole, encompassing dynamic and adaptive patterns, described by the term “synecticity.” It is stipulated that synecticity, along with the functional qualities of an events network, lie at the core of portfolio management enabling the interconnection of social networks and the community through the array of events.

## Grounding Presumptions of Event Portfolio and Its Social Value

### Conceptual Borderlines: Event Portfolio Perspective and Financial Theory

Any effort to build theory on event portfolios should consider and clarify, in conceptual terms, its affinity to financial portfolio theory in order to clear possible confusion over their meaning and application. At first sight, the event portfolio perspective resembles Markowitz's (1952) modern portfolio theory, but in essence, differs substantially since events constitute socio-cultural constructs that cannot be treated as financial assets (Ziakas, 2010, 2014a). Markowitz's portfolio theory stipulates how to make optimum investment decisions on financial assets that vary in terms of their anticipated return and risk. According to Markowitz, a portfolio is an assortment of financial assets (e.g., stocks, bonds, cash equivalents, and their exchange-traded counterparts). From this viewpoint, investors can benefit more from choosing portfolios that are diverse with a variety of assets enhancing their aggregate risk–reward value, rather than investing in single securities with seemingly high risk–reward traits. Diversification constitutes a fundamental tenet of financial portfolio theory functioning as a risk management technique. Diversification logics direct to combine a range of investments in a portfolio assuming that various types of investments can produce higher profits and face a lower risk than any single investment. Accordingly, the shared ground between financial portfolio theory and event portfolios is as follows: a mixture of events can enable more returns than single events, and events diversification can lessen the risk of not obtaining sufficient numbers of target audiences, thereby contributing to the attainment of comprehensive portfolio objectives (Ziakas, 2014a).

Separating the event portfolio perspective from financial economics, while also recognizing their common ground, helps disentangle and delimit the innate complexity of event portfolio management as a new art. It sets free this novel outlook to take shape through a realist managerialism angle and integrate resourcefully the range of events, approaches and policies under a joint ever-evolving framework. It also brings forward the conceptual roots, theoretical logics, and comprehensive foundations of event portfolio. The set of underlying ideas that have formed to date an embryonic event portfolio theory are outlined below focusing on its basic tenets, empirical origins as well as the conceptual framework of “Event Portfolio as a Multipurpose Development Tool” (Ziakas and Costa, 2011b) and the attendant model of “Synergistic Dynamics in an Event Portfolio” (Ziakas, 2013).

### Tenets of Event Portfolio Theory

Early conceptualizations of event portfolios (Jago et al., 2003; Chalip and Costa, 2005; Getz, 2005) were purely grounded in financial economics and business marketing approaching the phenomenon in a way similar to how a company manages its line of products (Getz, 2008). This business perspective dictated the treatment of events primarily as financial assets and commodities, implying the subordination of socio-cultural dimensions to economic imperatives, which as noted above, eventually create an imbalance over priorities within a portfolio context. In contrast, a broader conceptualization has been put forward intended to balance the economic-business and socio-cultural frictions among diverse events when their summative value is combined to shape portfolios as a multipurpose policy instrument (Ziakas and Costa, 2011b). Accordingly, the following definition of an event portfolio has been provided:

An event portfolio is the strategic patterning of disparate but interrelated events taking place during the course of a year in a host community that as a whole is intended to achieve multiple outcomes through the implementation of joint event strategies (Ziakas, 2014a, p. 14).

This conceptual delimitation points out that the development of an event portfolio is a strategic endeavor. It adopts a leveraging mindset to assemble various kinds of events in the quest for realization and amplification of certain aims. Events part of a portfolio can be recurring/periodic or occasional peripatetic one-off events that differ in terms of type and scale. As a result, the definition accentuates the importance of encompassing diverse event types of different magnitude so as to reach a varied spectrum of target markets. Diversification of events is important, but not an end in itself; instead, it should capitalize on the ability of each particular event to maximize the aggregate value of the portfolio. From this perspective, in order to exploit the full potential of portfolios, one must nurture synergies among ostensibly disparate events in ways that can enhance the role of each event for contributing to the overall value of the portfolio.

The concepts of relatedness and multiplicity are the epicenter of this portfolio definition. Portfolio relatedness denotes how events complement one another. Complementarity derives from utilizing know-how capacity, joint theming, resources, volunteer pools, or markets that might be created and retained by the range of events. Portfolio multiplicity denotes its ability to produce and express multiple meanings and attend multiple ends. To do so, joint event strategies should be implemented through cross-leveraging events with one another. The rationale of this definition is intended to facilitate the building of synergies among events based on the conceptualization of event portfolios as versatile devices that can be strategically leveraged for multipurpose development.

Along these lines, the perspective of event leverage provides a robust foundation for theorizing event portfolios because it dictates a focus on relationships amongst various events and/or their stakeholders in pursuit of achieving event outcomes. In this fashion, the aim is to cross-leverage events with each other and with the mix of local products/services in order to obtain and optimize compound benefits. To this end, it is important to grasp how events supplement one another, and then, how they may be supplemented by the local products/services of a host community. On the whole, cross-leverage is focused on grasping interconnections, nurturing synergies, and strengthening supplementarities. This comprehensive and holistically integrative approach can enable the formulation and implementation of joint strategies and ultimately help find the best means to leverage event portfolios.

### Origins of Early Empirical Evidence: The Fort Stockton Studies

The holistic approach on event portfolio was formed by a series of articles that investigated the array of events in Fort Stockton, a small agrarian community in Texas, *via* ethnographic doctorate research (Ziakas, 2007). The first article (Ziakas, 2010) demonstrated the operational base of an event portfolio by revealing the interconnections and synergies that accumulate various events as valued community assets and cultivate an integrative outlook for their utilization. The second article (Ziakas and Costa, 2011a) examined the logics and factors determining the utilization of an event portfolio as a tool for regional development. This study illustrated the necessity for combining event portfolios with local products/services and improving residents' quality of life. The third article (Ziakas, 2013) employed a dramatological lens that viewed events as dramatic narratives that embody varieties of a community's social order. Through this lens, the study showed that an event portfolio synthesizes a symbolic social framework, able to express different aspects of community life. Findings provided also evidence that events are interconnected both thematically and operationally. Based on this evidence, a multidimensional model was put forward that integrated the contextual, operational, and sociocultural facets of event portfolios. This work also supported another theoretical paper on event portfolios (Ziakas, 2014b) that offered a thorough perspective for achieving integrated strategic planning on the grounds of a policy community capitalizing on events and the synergy of its underlying inter-organizational network of stakeholders. In doing so, two integrative frameworks were proposed. One elaborating on event portfolio planning and leveraging, while the second concentrating on events networks and inter-organizational linkages. All this work was comprehensively presented in the ensuing monograph “Event Portfolio Planning and Management: A Holistic Approach” (Ziakas, 2014a).

### Multipurpose Development and Multidimensional Dynamics: A Comprehensive Framework for Event Portfolio Research

The work by Ziakas and Costa (2011b) offered an integrative conceptual framework that established the theoretical foundations of event portfolio as a multipurpose developmental tool. In this framework, an event portfolio is regarded as both a concrete and symbolic space formed by the interplay of formal-structural (*via* an events network) and informal (across and between social networks) relationships, event meanings, impacts, and community reactions, which, in turn, are affected by event implementations and their consequent outcomes. The framework sets forth that formal and informal interconnections impact in tandem the event portfolio as much explicitly as implicitly. The implicit impact is exhibited in public discourse and widespread attitudes toward events. The explicit impact is clear in the domain of policymakers who, acting in response to community issues, craft policies that define event roles and objectives.

The framework postulates that providing there is authentic representation of diverse matters, values, interests and attendant event meanings, a synergistic grounding logic can be grown incorporating an event portfolio into the structures and processes of the host community. This grounding logic can fortify the social and human capital created in events and configure their relatedness. In effect, the dynamics of this synergistic route may establish the sustainability of the event portfolio and attendant community capacity-building efforts. This can be done mainly by enabling the utilization of the requisite stakeholders, resources and community support toward coordinating, and cross-leveraging events to achieve portfolio goals.

In further analyzing the dynamics that can enable synergies across a portfolio, a multidimensional model was developed (Ziakas, 2013). According to this conceptualization, the principal task in managing event portfolios is to nurture synergies among dissimilar events and generate favorable conditions to cross-leverage these events for several gains. Thus, the aptitude of a portfolio as a policy instrument rests on its ability to produce various meanings and serve various aims. However, this does not imply that novel meanings and aims need to be put in place all the time, but that instead, managers should choose the optimum assortment of meanings and aims to tackle community matters and fulfill the portfolio's strategic goals. In addition, event portfolio managers must think of creative ways to link events both conceptually and functionally, hence cultivating their interconnections, which encompass the contextual, operational, and sociocultural grounds lying underneath the portfolio. These grounds are explained below with particular reference on how they make up a multidimensional context.

In particular, the model suggests that the conceptual interconnections stand for the sociocultural ground of an event portfolio encircling and embodying several resident standpoints through event meanings and symbolisms. This is described as “conceptual connectivity.” Based on anthropological scholarship (Turner, 1974; Handelman, 1990), the model postulates that the conceptual connectivity of event meanings is enabled by public discourse. Such discourse is metaphoric, conveying the dialectical expression of ontological issues that concern people. Conceptual connectivity is also enabled by the complementary and episodic sequencing of events as dramatic narratives. In this sense, the model asserts that the multiple forms of diverse events need to be symbiotic (as subject to portfolio design) connecting divergent varieties of social order, as epitomized by event themes, symbols, narratives, and meanings, into an integrative context. In this fashion, the conceptual connectivity can enhance the operational ground by gathering and combining disparate events.

Further, the model shows that the functional or instrumental interconnections stand for the contextual ground of an event portfolio containing the policy background, market/economic conditions, events network, resource capacity, and community traits/idiosyncrasies that have an effect on the delivery of the portfolio. This is named “instrumental connectivity.” It is posited that the instrumental connectivity of event purposes is enabled by an imperative common sense of purpose that brings together policymakers and event stakeholders to work on, as well as the ability of tapping into an integrated set of resources to deploy in event portfolio delivery. On this basis, the instrumental connectivity can enhance the operational ground by driving cooperation and resource-sharing among the network of main local actors and event stakeholders.

Along these lines, the model puts forward that event relatedness is built by the multiplicity of event meanings and purposes, and the nucleus of their conceptual and instrumental connectivity. In this respect, events may supplement each other *via* their thematic emphasis that bolsters deliberate meanings, utilization of shared volunteer pools that assist in the staging of events, transfer of tacit/proprietary knowledge and experiential capacity across locals in event management, and investment in reaching new or accompanying markets. According to this model, the synergistic outlook in the planning of event portfolios involves principally their design practices regarding what events must be included in the portfolio and their reach, frequency, timely placement, and fit, as well as the size of the portfolio (number of events). In short, although the model sheds light on the synergistic mechanics that give shape to portfolios, it remains unclear how their social value can be optimized.

### The Social Value of Event Portfolios: Improving Community Quality of Life

The growth of event portfolios is innately interlinked with the strengthening of local social networks, being dependent upon them, and then enhancing the social capital generated and sustained through the array of events (Ziakas, 2014a). This brings forward the potential of portfolios to strengthen local communities and better their quality of life. Indeed, Richards (2017a) notes the changing use of events by cities from a chiefly place-branding role centered on image and economic impacts toward a wide-ranging placemaking approach intended to achieve holistic improvements in place quality and destination attractiveness. Likewise, Westerbeek and Linley (2012) argue that cities hosting event portfolios are more likely to be seen as destinations with finer quality of life, and as therefore, more appealing to live and work in. Moreover, Dragin-Jensen et al. (2016) found that portfolios with a composition of few major, but mostly international top-events, are more likely to attract new residents than portfolios with diverse, but mostly local and non-top-events, because the former offer higher levels of perceived variety in life. In addition, the same authors found that portfolios focusing on few major events have stronger effects for residents living in large cities, and that the type of events offered in the portfolio such as sport or cultural events does not make a significant difference.

Another study by Antchak (2017) on Auckland's portfolio, demonstrated an outcomes-driven approach emphasizing on major sport events that align with the city's policy to become liveable, and thus, enhance its attractiveness for tourists, businesses and investors. However, such dependence on external event markets overshadows local needs and demand, hence, limiting the portfolio social benefits. Instead, portfolios can be leveraged to foster their social value by satisfying concurrently exogenous and endogenous markets, and complying with local capacities, physiognomies, resources and needs so that they improve residents' quality of life (Ziakas and Costa, 2011a; Gibson et al., 2012; Taks, 2013; Getz, 2017). Wallstam et al. (2020) in their search for unified indicators of evaluating the social impacts of event portfolios suggested the following set of indicators: (1) community quality of life, (2) community pride, (3) social capital, (4) sense of community, (5) community capacity enhancement, and (6) facilities impact.

The literature has recognized that the capacity of event portfolios as a strategic policy device is considerably versatile being reliant on local needs and conditions (Ziakas and Costa, 2011a; Antchak and Pernecky, 2017; Antchak et al., 2021). Several examples have been studied and documented. Specifically, in the college-town of Gainesville, Florida, local conditions favored the development of a small-scale sport event portfolio to promote sustainable tourism (Gibson et al., 2012), whereas in London, Ontario they facilitated the assemblage of sport “ice” events as a means for urban development (Clark and Misener, 2015). Also, in a popular Portuguese resort, local conditions enabled the formation of a portfolio consisting of coastal sport events, which helped the resort to create its nautical destination brand **(**Pereira et al., 2015). Equally, it has been pinpointed that agrarian communities can utilize an event portfolio for accomplishing regional development (Ziakas and Costa, 2011a), tourism repositioning (Presenza and Sheehan, 2013), and post-disaster recovery (Sanders et al., 2015). Besides, it has been shown that portfolios can be harnessed for historic commemoration (Viol et al., 2018), place branding (Westerbeek and Linley, 2012; Andersson et al., 2020), and flow-on tourism (Taks et al., 2009). Correspondingly, findings substantiate the claim that portfolios yield significant tourism revenues (Salgado-Barandela et al., 2021) as they increase visitor satisfaction and expenditure (Buning et al., 2016; Almeida et al., 2019; Almeida and Garrod, 2021). Concomitantly, evidence indicates the portfolio value for residents by enhancing, for instance, local quality of life for families (Booth and Cameron, 2020). By the same token, the potential of portfolios has been suggested to leverage the Olympic heritage for sport and cultural tourism (Boukas et al., 2013), and to diversify island tourism (Ziakas and Boukas, 2016).

In the literature, there is indication that a community-based portfolio is contingent upon locals' attitudes and opinions about event tourism development. This seems to be the case because of the evidenced robust association between locals' attitudes toward tourism development and their perceptions of their extent of participation in the crafting of strategy and trajectory of development (Presenza and Sheehan, 2013). Consequently, it is crucial to involve locals in the strategic planning of event portfolios *via* using an inclusive and democratic participatory planning methodology. Portfolio governance, hence, needs to be viewed as a communal space for leveraging the produced social capital, wherein stakeholders negotiate their individual interests and take collective action to attain shared goals. In so doing, understanding locals' attitudes toward an event portfolio constitutes a very useful preparatory process for policy, planning and strategy. As demonstrated in the instance of the Sunshine Coast's portfolio in Australia, local support for event tourism strategies can be elevated when it is sensed by residents that they gain from magnifying the joint use of events, venues and occasions to both attend and participate, keeping costs low, supporting family-oriented festivals, and creating hallmark events (Gration et al., 2016). Therefore, strategic portfolio planning entails making sense of how locals value events by connecting impact assessment to resident perceptions and attitudes toward events and their valuation.

Recent scholarship has also begun to shed light on strategic portfolio leveraging for creating community value. Pereira et al. (2015) set forth as major planning factors for effectual leveraging the appointment of a local committee in charge of the events and the multiplicity of benefits obtained by one joint strategy. The latter entails to cross-leverage a series of events for various ends. As demonstrated by Pereira et al., in the study of a confined nautical portfolio, an overarching vision was missing to meticulously advance events' synergies and to augment their supplementarities, therefore ending up in unexploited opportunities for cross-leverage. Contrariwise, Kelly and Fairley (2018) recorded how the founding of an Event Board succeeded to generate and ease relationships amongst stakeholders from tourism, events, and government who entered to administer the growth of an event portfolio.

Consequently, planning to form apposite conditions for networking and relationship-building is crucial for the efficacy of portfolio leveraging (Chalip, 2004; Ziakas and Costa, 2010a; Ziakas, 2014a). This involves establishing a local coordinating unit to monitor portfolio delivery and create a fertile ground for stakeholder participation, communication, and dialogue. For instance, in the city of Edinburgh, the founding of Festivals Edinburgh as a strategic umbrella agency has been central in the remarkable growth of its portfolio by representing several festivals jointly and implementing cooperative projects (Todd et al., 2017). Hence, relationship and network capacity can be fostered in order for solidifying cooperation within a portfolio, which is pivotal in its operation and administration (Dickson et al., 2018). In effect, the attainment of multiple leveraging goals within a portfolio requires the coordination of the network of organizations part of the events, which include local and external actors (Pereira et al., 2020). This means that the local coordinating entity should have mixed composition inviting also external stakeholders.

Early scholarly work contextualized the community and social value of portfolios as one that may shift cities from merely hosting events to become “eventful cities” (Richards and Palmer, 2010). This thesis is based on the notion of “eventfulness,” indicating that an “eventful city” adopts a strategic, holistic vision of its event portfolio (Richards and Palmer, 2010; Richards, 2017a). From this perspective, Richards (2017b) scrutinized the ways several cities are creating more holistic approaches to event strategy and eventfulness. Further, Getz (2017) considered the intersection of portfolios with sustainability suggesting that its quest in eventful cities requires oversee overlapping portfolios of events, presume a long-run perspective, and allow for compound takes on value and aggregate impacts. Consequently, the extrinsic (return on investment) and intrinsic (socio-cultural) values were revealed as dualistic angles of value that ought to be combined for yielding the cumulative portfolio value. Another immanent dualism was observed by Richards (2015) concerning the distinction between iterative and pulsar events. Richards (2015) explored a far-reaching part of events as being social agents with the capacity to both maintain and change social systems. This analysis illustrated that the preservation of social systems is contingent on iterative events (i.e., recurrent events re-validating social structures), while pulsar events (i.e., one-off mega-events) may metamorphosize social structures. Richards' research brings forward the prospective interaction amongst various kinds of events within a portfolio, which can prompt the continuation of the social order *via* iterative events and its metamorphosis *via* pulsar events.

From a strategic planning standpoint, the generation of social value through portfolios involves a number of processes. At first, it is pivotal to create conditions for garnering wide community support and volunteerism, handling opposition, and fostering collaboration between local stakeholder groups. These conditions are necessary for the strategic leveraging of events aimed to maximize their impacts to host communities (Chalip, 2004; O'Brien, 2006). Thus, it is essential to activate the entire community encouraging wide involvement and enabling inclusive participation. Accordingly, there is a growing recognition for the need to implement participatory planning, and thereby, allow input from different stakeholders. Engagement and participation in portfolio planning requires building the capacity of a host community to enhance and effectively use local skills, knowledge, confidence and structures in undertaking community development initiatives (Frank and Smith, 1999; Noya et al., 2009). Such capacity means that a host community is able to understand emerging or diachronic needs and problems, and find the means to address them through implementing appropriate strategies.

## Setting the Terrain for Community Portfolio Capacity-Building: Residents' Engagement and Participatory Planning

The concept of community capacity-building is a relatively new addition to the policy lexicon that has been used since the 1990's primarily in the fields of health, education, agriculture, and community development (Craig, 2007). Although it is widely accepted as a resilient strategy to improve the well-being of individuals, groups and communities, there are different conceptualizations in the literature seeking to encompass and explain the complex system of processes and interactions that shape community capacity-building (Frank and Smith, 1999; Noya et al., 2009). For example, Goodman et al. (1998) defined community capacity as a process and outcome including supportive organizational structures and processes, which operates at the individual, group, organizational, and community levels within a specific context. Community capacity has also been conceptualized as the levels of competence, ability and skills necessary to set and achieve relevant goals (Balint, 2006). Another definition of community capacity describes the concept as including the assets and attributes that a community is able to draw upon in order to improve their lives (Laverack, 2006).

These different conceptualizations reflect the intangible nature of community capacity and the subsequent difficulty to measure it. Yet, they are based on the common ground of social capital and social integration. Accordingly, a comprehensive conceptualization has been provided by Chaskin et al. (2001) who view community capacity as the interaction of human, organizational and social capital existing within a community that can be leveraged to solve collective problems and improve or maintain its well-being. On this basis, they developed a relational framework for understanding community capacity-building that takes into account the informal social processes and/or organized efforts by individuals, organizations and networks incorporating in addition attempts/strategies to build community capacity, the influence of context (i.e., conditioning influences that support or inhibit capacity-building) and suppositions about community-level outcomes. The framework suggests that community capacity is exemplified by a set of characteristics and operates through the agency of individuals, organizations, and networks (levels) to perform particular functions such as informing, organizing, and mobilizing residents toward collective action. Four fundamental characteristics of community capacity are suggested: (1) sense of community, (2) level of commitment among community members, (3) ability to solve problems, and (4) access to resources. Further, the influence of context (i.e., conditioning influences that support or inhibit capacity-building) and suppositions about community-level outcomes are also emphasized (Chaskin et al., 2001).

The characteristics, functions and conditioning influences of this framework seem to be consistently related to resident attitudes, community participation in, and support for event portfolios. Specifically, the sense of community reflecting a degree of connectedness among members, including collective values, norms, and vision (Chaskin, 2001) coincides with the conceptual and instrumental connectivity among events. This is also associated with locals' level of commitment, which describes the responsibility that individuals, groups, and organizations take for what happens in the community (Chaskin, 2001), and the levels of trust in local institutions that form social capital and support for an event (Arai and Pedlar, 2003; Misener and Mason, 2006a). Moreover, the ability to solve problems by translating commitment into action and the access to economic, human, physical, and political resources (Chaskin, 2001), are inextricably connected with inclusive community participation in portfolio management. As such, the community capacity function of planning, decision-making, and governance (Chaskin, 2001) can be enabled by the empowerment of locals to participate in all stages of portfolio planning and delivery. Conditioning influences of community capacity such as safety or economic opportunity can be described as the existing contextual conditions of residents' quality of life and their expectation to be improved by the array of events.

While there is a paucity of research on community capacity-building for events and portfolios, some evidence was offered by VanWynsberghe et al. (2011) who examined community capacity in the case of the Vancouver 2010 Winter Olympic Games, scrutinizing how a community-based coalition was established to monitor the sustainability mandate of the Games. Specifically, this study investigated the conditions for capacity-building in the staging of this mega-event and its impact on activating the potential of the host community to recognize, address and resolve serious public concerns. Although the study concluded that capacity-building was eventually limited due to lack of resources or recourse, findings evidenced the characteristics and conditions that foster its development. As such, key means to building event capacity are solid legitimacy of event-related organizations or coalitions for decoding public concerns into beneficial outcomes, implementing inventive methods for involving the community, and deploying available resources, skills, experience, know-how and leadership to plan sustainable development activities. According to this study, community capacity-building can be understood as a continuing, cyclical and iterative interchange among individuals, organizations and community that hence encompasses the dimensions of context, resources, activities, and outcomes, and the levels of individual, organizational and community. This study focused on organizational level arguing that agencies are the most effective enabler of community capacity because they are the principal channels whereby individuals and networks implement their activities. From this standpoint, it is highlighted that as agencies deploy resources to execute actions, they seek out to realize outcomes meeting their goals. Hence, the operational basis of capacity-building can be better understood on the organizational level by shifting the focus from possessing the capacity to expending these skills, resources and knowledge through collaboration.

The value of collaboration is pivotal for social organization and integration and is well-documented in community tourism planning (Jamal and Getz, 1995; Bramwell and Sharman, 1999; Hall, 1999), while it remains relatively under-examined in event studies (Larson, 2009; Yaghmour and Scott, 2009; Adongo and Kim, 2018). Individual attitudes of members play an important role as collaboration requires skills and positive attitudes/behaviors. Since events in a portfolio rely substantially on volunteer members, they can be easily influenced by the wider resident perceptions about them. Thus, individuals' attitudes, participation in, and support for a portfolio constitute the preconditions or lifeblood of community capacity-building that need to be thoroughly understood in order to be effectively leveraged.

Within a community capacity perspective, quality of life is central. In particular, community capacity is viewed in the community development literature as the set of assets or strengths that residents individually and collectively bring to the cause of improving local quality of life (Simpson et al., 2003). Also, existing standards of quality of life are essentially a conditioning influence on community capacity such as residential stability that enhances social networks, supporting a sense of social cohesion and the likelihood of participation in local activities (Chaskin, 2001). Quality of life is a multidimensional concept composed of socially- and culturally-related factors such as life satisfaction or happiness, and includes both objective (i.e., conditions of life) and subjective (i.e., experiences of life) dimensions (Kaplanidou et al., 2013). Research suggests that single events and portfolios bring about substantial improvements in residents' quality of life (Liburd and Derkzen, 2009; Ziakas and Costa, 2011a). For mega-events, Karadakis and Kaplanidou (2012) found that Vancouver residents of the 2010 Winter Olympics valued economic, environmental, infrastructural, and socio-cultural legacies for their quality of life. By extension, Kaplanidou et al. (2013) examined the mediating role between quality of life impacts and residents' support for hosting the 2010 FIFA World-Cup showing that positive perceptions of event impacts on quality of life led to increased support. These findings corroborate previous research, which found the importance of intangible mega-event impacts such as improved socioeconomic conditions (Preuss and Solberg, 2006; Kaplanidou, 2012). Portfolios represent a more intricate context where interactions of large-scale events with smaller events may engender mixed implications for quality of life and the engagement of social networks. For example, antithetical values and interests due to the scale of events may create antagonism and conflict among different community groups, thereby dividing and disengaging social networks from supporting a portfolio.

Notwithstanding the evident intricacy of portfolios, current research fails to account for their political dimensions affecting community quality of life. The underlying force for driving destinations to adopt deliberate strategies and make the most of events is underpinned by the neoliberal, entrepreneurial governance (Burbank et al., 2002; Foley et al., 2011; Hall, 2012). This ideological proclivity frames event policy aims to chiefly align them with destination branding and economic impact imperatives, at the same time as incentivizing private sector involvement (Foley et al., 2011). Portfolio development faces the perils linked to a vastly entrepreneurial event governance, including inequality, marginalization, and social polarization (Foley et al., 2011), as elites with better access to resources and capital may profit at the cost of disadvantaged parties (Ziakas, 2015). Obviously, these risks significantly hinder the social value of portfolios and deteriorate community quality of life.

To confront this challenge, stakeholder inclusiveness and participation in the planning, management and governance of portfolios is necessary so as to enable equivalent distribution of effects and gains (Ziakas, 2015). Such an inclusive approach demands the founding of an open, long-standing, and accountable system wherein bottom-up planning and strategy implementation takes place *via* the involvement and active support of residents in event structures and decision-making (Misener and Mason, 2006b; VanWynsberghe et al., 2011; Jepson et al., 2013). Nevertheless, there is a scarcity of scholarship on event governance and participatory planning. On the other hand, the highly centralized and formalized context of large-scale sport events, controlled by event owners and global conglomerate networks, shows the dominance of top-down decision-making in event management with power and authority be vested merely in chief executives commanding henceforth the distribution of gains (Roche, 1994; Horne, 2007; Ziakas and Boukas, 2012).

A noteworthy exception is the investigation of the 2009 Australian World Rally Championship by Dredge and Whitford (2011) who scrutinized how event governance is being molded by the public sphere as a nascent type of public–private policymaking. The public sphere comprises the space of dialogue and participation in which stakeholders deliberate on and move forward undertaking activities that attain collective objectives. Habermas (1989) viewed the public sphere as a theater of democracy, a space for the enactment of political participation. He brought into focus the blurring of public-private interests, stressing that private interests find their way into public debates and are expressed by representing public interests. In this manner, the public and private life are converged since the emphasis of public dialogue is on an interchange of thoughts, views and logics rather than individual interests. Dredge and Whitford claimed that a discursive public sphere is appropriate for structuring the space of dialogue, interaction and information-sharing. This may ensure stakeholder inclusiveness and engagement in event portfolio planning and governance. Moreover, it could support the use of an asset-based community development perspective as a way to develop an action-oriented, community-based method to leveraging the social assets of events (Ziakas and Costa, 2010b, 2012; Misener and Schulenkorf, 2016). Overall, the fostering of an open dialogic space and participatory planning based on community inclusiveness, engagement, and empowerment can build a community's capacity to improve its conditions and quality of life. There is no systematic attempt yet in the literature to integrate single events and portfolios with the perspective of community capacity-building, leaving thus, a gap about how attitudes and support can be mobilized to enable collective action by leveraging the social capital generated in events (Ziakas, 2016).

Within event management scholarship, community capacity-building has been given limited attention, and consequently, little is known about how to build community capacity through single events or portfolios, and contribute to improving quality of life. Therefore, the extant literature on event management needs to expand the focus on community capacity-building and participatory strategic planning processes. To do so, a more nuanced understanding is needed of how the involvement of a host community's social networks affects support and capacity-building for event portfolio development.

## Building a General Explanatory Framework

Event portfolio theory and its relationship with community capacity-building have been portrayed in somewhat disjointed parts of the literature, which resembles more a mosaic of conceptual fragments. The danger is that event portfolios may end up being dividing, rather than integrative, phenomena as part of an over-fragmented event industry. So to draw together the different germane matters, parameters and themes into a coherent elaboration of how portfolios are interlinked with community networks, I provide a conceptual groundwork on the intersection between the factors that affect strategic portfolio planning and the dynamics that shape stakeholder attitudes, participation in, and community engagement with portfolios. The conceptual analysis includes theoretical elaborations regarding integrative strategic portfolio planning (Ziakas, 2014b), event portfolio network and community capacity-building (Ziakas, 2019b), portfolio tourism leveraging (Ziakas, 2020), and the undercurrents lying beneath attitudes, participation in, and community involvement in order to underpin the building of a broader framework for shaping event portfolios as social systems. By appreciating this multifactorial process as a synectic route, a common ground is offered for fostering and leveraging the interrelationships among stakeholder attitudes, inclusive engagement and participation, social capital and community capacity-building in the management of event portfolios. This is essentially what I call “morphosynthesis” of portfolios, a complex adaptive and integrative social process as the nature of portfolios is.

The perspective of morphosynthesis is grounded in part in Archer's (1995) morphogenetic approach as the prefix “morpho” exemplifies. Like in morphogenesis, with morphosynthesis I mean an explanatory framework accounting for the social dynamics and processes shaping event portfolios as systems through an analysis at all levels from the micro- to the macro-level. Thus, morphosynthesis seeks to uncover the generative mechanisms that influence the interplay among the elements of “structure,” “culture,” and “agency” in the configuration of event portfolios. Since this interplay is dependent upon local contexts and conditions, the resultant set of forces existent in each host community may create multifarious portfolio arrangements and formations. This kind of variety has the potential to induce further variety in terms of creating new knowledge, competencies and stakeholder relationships on managing a series of events through the connection of their previously unrelated but complementary cultural elements. However, the perspective of morphosynthesis deviates from morphogenesis as it gives emphasis on the process of “synthesis” positioning it as a focal point of portfolio management. The “synthesis” refers to the constant and adaptive integration of conceptual, contextual, policy and operational grounds of portfolios. This is also evident in the term “event portfolio,” which seeks to integrate the business and financial origins of “portfolio” with the nature of “events” as socio-cultural constructions. In the next sections, the pertinent theoretical elaborations on portfolios are discussed in order to underlie the formulation of morphosynthesis as a general explanatory framework for portfolio development and community capacity-building.

### Integrative Strategic Portfolio Planning

Former theoretical work examined practices, routes and means for integrative strategic planning of portfolios and assistant inter-organizational networks (Ziakas, 2014b). In this analysis, a framework was built distinguishing the main causes that affect event portfolio planning and leveraging, which include the institutional structures, patterns of social relations, local resources, and market demand. Especially, an institutional structure creates the formal organizational framework by which events are planned, delivered and leveraged. The patterns of social relations affect the informal ties of social networks and the nature of social exchanges that support collaboration and synergies. Local resources include the local community raw capitals (i.e., human, social, natural, financial, etc.) that can be utilized to enable event hosting and cross-leveraging. Market demand unveils the levels of interest in endogenous or exogenous target markets. Taking into account these major factors, host communities must craft joint event portfolio strategies attuned with their broad policy aims.

The same framework points to the policymaking conditions that enable the synergistic objectives and scope of an event portfolio. The common domain of intersecting tasks and shared benefits makes up an informal network that has an effect on portfolio planning. The policy universe is the overall number of actors and autonomous interest parties competing with one another for control over policy development. The interdependencies between event stakeholders and the policy community that is concerned with sectoral matters encompass the event policy network. The policy network is the connecting means emerging as the product of interactions within a policy community. An event policy network must regard a portfolio as a community asset with the potential to put in place joint strategic planning and nurture reciprocal relationships so as to attain compound policy ends. On these grounds, the integrated strategic planning aimed at leveraging an event portfolio can set up joint policy ends across different domains.

In terms of how integrated strategic planning can best be used, the framework posits that concerted portfolio delivery comprises a number of organizational antecedents and implementation parameters. The former are the following: (1) event-network embeddedness, (2) inter-organizational reciprocity, (3) event integrity, and (4) participatory inter-connectedness. With event-network embeddedness, the point is that an event policy network needs to hold solid, lasting relationships between event stakeholders and the policy universe. In this respect, embeddedness is understood as the extent to which portfolio actors are embedded into local economies *via* relationships with stakeholders. Thus, the concept concerns the intersection of social and economic linkages that affect event implementations and leveraging strategies, as well as the nesting of event-related ties within other social relationships. Accordingly, actors' behaviors are embedded to the degree that they lean to interact with associates who share mutual gains, or if simply their trade partners prefer to deal with one another. On the grounds that event agencies are embedded into the social structures, inter-organizational reciprocity can be enabled. In effect, a host community can attain the shared employment of resources, facilities, skills, knowledge, and human labor to support its portfolio. The enrichment of collaborative behaviors by the extant amounts of trust and reciprocity works out as a channel to produce social capital. Similarly, the event portfolio can be turned into a space for the production of social capital by nurturing relationships of trust, mutual recognition or obligation and kind aid among organizing entities. In this vein, inter-organizational connections must further collaboration and reciprocity in order to reinforce joint decision-making and problem-solving in event implementations and cross-leveraging strategies.

Regarding the integrity of an event portfolio, two dimensions are distinguished, that is internal and external. The concept, overall, concerns the consistency of all events in embodying authentic community ideals and meeting attendees' needs. The internal dimension requires that events have consistent socio-cultural fit with the host community. Internal integrity throughout the event portfolio may be reached principally *via* inter-organizational coordination within the event policy network. The external dimension of event integrity requires that event implementations consistently satisfy the anticipations of attendees. Maintaining portfolio integrity is related to participatory inter-connectedness of residents. The staging of periodic events may strengthen residents' sense of community and improve their quality of life in the provision that opportunities are designed across the portfolio for event attendees to undergo esoteric development and to (re)build shared identities *via* their energetic and reflexive participatory inter-connectedness with one another in events' performances. Relationship-building efforts must establish more profound social links, thereby contributing to the resuscitation and heightening of a host community's social capital. The beneficial effects of sociability can be magnified by boosting the participatory inter-connectedness all over events in a portfolio. For that reason, an event portfolio must offer plentiful chances for more unrestrained social interaction and the development of deeper social relations in which social capital can be produced.

Further, the framework puts forward a range of implementation parameters determining integrated strategic planning. They include the following: (1) utilitarian scope, (2) dialectical expressivity, (3) symbiotic polymorphism, and (4) resource inter-changeability. Utilitarian scope signifies the common sense of purpose that drives the devising of event leveraging strategies and legitimizes them in the public domain. It is a manifestation of the host communities' concerns and its deliberate attempt to exploit various events for a clear array of ends. Dialectical expressivity signifies the suite of symbolic meanings that are conveyed *via* the range of events on important issues and enable metaphoric public discourse. In other words, dialectical expressivity frames a metaphoric dialogue *via* a series of events. Symbiotic polymorphism signifies the multiformity of events in the portfolio, which connects various event elements, themes, and meaning patterns into a coherent whole. This is accomplished through common elements that are part of various events, which may enable continuities (of activities) amongst them, even though they are attracting varied target markets. Common elements may additionally fortify polysemy as they are part of a sequence of events and may assist in instantiating their shared meaning. Finally, resource inter-changeability signifies the capacity of tapping into a shared pool of resources for staging various events across the portfolio. This entails a conjoint appreciation of resource interdependencies, intersecting functions, interests, and needs existing in an event policy network in order to smooth the shared use of resources in a portfolio.

### Event Portfolio Network and Community Capacity-Building

In the aforesaid analysis, the concept of an events network is distinguished from a policy network, and defined as:

An events network is the non-institutionalized array of organizations that make decisions and take actions regarding planning and implementation of events in a host community as well as tend to engage in relationships that facilitate their goals. To the extent that collaboration patterns among the participating organizations are characterized by reciprocity and trust, synergies among events can be developed and strengthened within the portfolio in order to maximize the benefits attained for the host community (Ziakas and Costa, 2011b, p. 414).

According to this definition, an events network is an informal group of organizations that are integral to the portfolio processes and create informal relationships with other actors of that group so as to attain their own aims. This conceptualization of an event portfolio network is pivotal for achieving integration across the leisure, culture, sport and tourism domains, because they function in an invisible network of actors who engage in various kinds of relationships in order to deliver their services. Moreover, the definition makes clear that an events network is a serendipitous system permeated by individual interests, which hinder the means of forming linkages. As a result, event portfolio networks have predominantly decentralized structure and rest on dyadic relationships characterized by interpersonal trust. Collaboration offers channels by which resources flow, thereby forming the whole system of event organizations within the portfolio.

The conceptualization of an events network served as a basis of the resultant model delineating the event portfolio network as a mechanism that can be measured and assessed (Ziakas, 2014b). According to this model, inter-organizational collaboration within a portfolio's events network largely occurs through information exchange, resource-sharing, joint initiatives and joint problem-solving that can eventually build community capacity to deliver events effectively and efficiently. These relationships epitomize links correspondingly that can be computed to illuminate inter-organizational connections and collaboration forms. On these grounds, inter-organizational collaboration forms can be evaluated by calculating the reciprocity and trust of collaborating agencies. Considering that relationships are not static, but rather continually transforming and evolving, this model can be employed to assess the status of the collaborating network across time and different conditions. All in all, this model provides an applied method to elucidate the inter-organizational relationships that strengthen the capacity of a community to manage and leverage an event portfolio.

In bringing together the events network model with a holistic perspective for planning, managing and leveraging event portfolios, a functional framework for building community portfolio capacity was put forward (Ziakas, 2019b). In this framework, portfolio planning has at its center the community policy goals that define event purposes, utilize event infrastructure as an integrated set of resources, formulate leveraging actions, and establish operational mechanisms to achieve sustainability. The framework illustrates that event stakeholder dealings and interchanges configure a collaborative network working together in event implementations across the portfolio. To this end, the framework supports that effective collaboration can be reinforced by augmenting norms of reciprocity and trust in information exchange, resource-sharing, joint initiatives and joint problem-solving. It is suggested that such robust stakeholder connections can enable portfolio coordination and build community capacity in portfolio management and leveraging, thereby attaining desirable outcomes. Lastly, the framework concludes that in order to optimize and sustain event outcomes, holistic portfolio evaluation should be conducted to update and adjust portfolio planning, and therefore, assist its sustainable growth.

### Portfolio Tourism Leveraging

The processes of portfolio tourism leveraging are scrutinized in recent work (Ziakas, 2020), which suggests as its basis the suite of interrelated destination capitals, including socio-cultural, human, environmental, financial, political, and technological capitals. The critical issue is how they can best be cross-leveraged with the event portfolio so as to magnify sustainable tourism gains for the host destination. As already highlighted, in the planning phase of portfolio leveraging, it is critical that a discursive public sphere is created in which the various stakeholders are engaged in dialogue deliberating on and taking action to attain common goals. A discursive public sphere can enable inclusive stakeholder participation in portfolio planning and governance, if an open accountable system is instituted, which permits the committed involvement of residents in all phases of portfolio decision-making. This kind of a participatory planning scheme must be able to make certain the coordination of cross-leveraging strategies between event and destination organizations, and enable the even distribution of gains amongst destination stakeholders. Portfolio planning and governance should be overseen and coordinated by a local entity charged with the mission of forging the requisite linkages and coalitions for effective portfolio tourism leveraging. A primary responsibility for the coordinating body is the design of the portfolio, which comprises decision-making about the reach and frequency of various events, their timely placement (scheduling), their fit amongst them and with the destination, as well as entire portfolio size. The design approach molds the composition of the portfolio in accordance with exogenous demand and endogenous destination conditions, assets, and operational requirements.

The above interconnected components create the conjoint terrain of portfolio planning, governance and design that realizes its tourism leveraging. To execute effective leverage, the portfolio should be aligned with the general placemaking policy of the destination and the utilization of the supporting events network because both affect substantively the crafting and application of tourism leveraging strategies. As such, an event portfolio should be seen as a leverageable resource that brings the opportunity for tourism leverage of event visitors and destination assets. This opportunity necessitates joint strategic planning between the portfolio coordinating entity and the destination, which can be enabled with the following set of strategies: (1) Amplify visitation, (2) Diversify tourism product, (3) Extend the tourism season, (4) Foster event tourism networks, (5) Consolidate destination assets, and (6) Bolster destination's authenticity. To magnify portfolio outcomes, this set of leveraging strategies must be conjointly executed and synchronized. The *ad-hoc* application of only some strategies would not enable optimum portfolio leverage.

In turn, the outcomes and returns that derive from the execution of the above set of strategies should be assessed *via* a comprehensive cost-benefit analysis compared to a triple-bottom-line framework of social, economic and environmental effects, and their subsequent role in tourism development. The findings of evaluation should report how well destination capitals are cross-leveraged with the portfolio. In the end, a review of the effects of leveraging activities on the state of the destination's productive assets is needed so as to appraise their synergy with the tourism placemaking policy, and accordingly introduce modifications. These steps overall indicate the resonance of synchronous cross-leverage, formative and summative evaluation, resultant learning, knowledge exchange, and information-sharing across portfolio tourism leveraging activities. As such, it is essential to enable strategy synchronicity and utilize feedback loops in portfolio delivery and leveraging.

### Dynamics of Event Stakeholder Attitudes, Participation, and Community Capacity

To look at the dynamics that are intertwined with capacity-building and shape stakeholder attitudes, participation in, and community engagement with portfolios, we need to review the literature on single events as there is scant research on this area about portfolios. This literature brings to the fore as critical the factors of power, community participation, and public (or institutional) trust.

Events are avowedly sites of power since they are staged through stakeholder interactions and exchanges as each actor is trying to have influence over the planning and obtain benefits (Parent, 2010; Dredge and Whitford, 2011). Power, thus, is central in event planning determining resource allocation, strategy formulation and distribution of benefits. Although large-scale events have traditionally followed top-down centralized models, there is recently recognition that participatory planning can engage and empower residents to partake in event planning and decision-making, enabling thus the active involvement of stakeholders (VanWynsberghe et al., 2011). Jepson et al. (2013) found that residents' attitudes are positively influenced by the perception of successful participation, whether they felt that their views are considered within the planning process and the knowledge gained.

Participation in events can foster interpersonal connections enhancing social cohesion, trust, mutuality, cooperation, and openness (Arai and Pedlar, 2003; Moscardo, 2008a,b; Jepson et al., 2019). Additionally, distinctive event networks can take shape from the recurrent development of these interpersonal connections in the delivery and promotion of community events (Mackellar and Jamieson, 2015). Therefore, opportunities for different stakeholders to participate in planning, such as their inclusion in the events network activities, can enhance their trust to event-related entities (Ziakas and Costa, 2010a), and establish positive attitudes and support toward an event (Van der Steen and Richards, 2021). This contributes to community capacity by building the skills and knowledge, leadership, and a sense of efficacy for dealing with problems (Simpson et al., 2003; VanWynsberghe et al., 2011). In effect, residents' attachment to the host community can be enhanced by fostering a heightened sense of community and commitment that drives levels of agency to take action and solve community problems (Chaskin, 2001; VanWynsberghe et al., 2011).

A critical factor in building community capacity is trust toward civic organizations. Such public trust in institutions is the confidence that institutions would not misuse power, and hence, it is a basic precondition for establishing cooperation between two parties, garnering support for development policies, improving resident attitudes toward government outputs, and strengthening people's confidence in government decisions (Nunkoo and Ramkissoon, 2011). Lack of trust in institutions can have reverse results and increase opposition against development programs (Nunkoo and Ramkissoon, 2011). Likewise, institutional trust is necessary to legitimize events in the bidding stage and garner community support in expectation that the event will create positive legacies (Preuss, 2007). Further, the building of trust is essential to engage stakeholders in exchange relationships and collaboration toward achieving the event objectives (Karlsen and Nordström, 2009; Yaghmour and Scott, 2009). In contrast, lack of trust in event-related entities decreases interest of residents in participating and their support for the event or it even may create resentment when event organizing committees are embroiled in corruption scandals (Mason et al., 2006).

In fact, there is a suspicion about events when they are controlled by elites that they are mandated to serve their interests at the expense of weaker groups (Rojek, 2013). For example, the public subsidies are justified on anticipations for producing economic impact (Burbank et al., 2002), which nonetheless is not often attained, and therefore, pushes host communities to realize in retrospect the exaggerated benefits and underestimated costs of an event (Whitson and Horne, 2006; Horne, 2007; Rocha, 2020), thereby eventually decreasing public trust over event-related institutions. Inclusive participation of residents in event planning may bring transparency and enhance public trust in events as they engage in exchange relationships in which although personal concerns act as guides of their actions, they are eventually determined and realized through reflexive deliberation. Relevant literature supports that trust is a product of various exchange relationships (Lioukas and Reuer, 2015) and a means to foster social capital (Podolny and Page, 1998). This is critical for building community capacity based on norms of trust and reciprocity, social networks and a culture of openness and learning (Simpson et al., 2003). By extrapolation, it can be argued that residents' trust in event-related institutions is likely to influence attitudes and participation in an event.

Community participation is based on bottom-up approaches that permit policies to be more socially-inclusive and help ensure the social stability and cohesion without which economic growth and structural adjustment will be obstructed; such approaches require a process that builds on local strengths and promotes community participation, leadership and ownership of both the problems and the solutions (Simpson et al., 2003). Consequently, the focus of capacity-building initiatives rests on facilitating resident participation in organizational decision-making and mobilization, and enabling problem-solving through empowerment and access to resources (Chaskin, 2001). When residents participate and interact in community-based joint projects, their dedication and support for the event and acceptance of associated development initiatives is strengthened (Schulenkorf, 2012). On the whole, residents' participation in events is beneficial in terms of sustainability and effectiveness of the implemented actions (Quinn, 2009; Lamberti et al., 2011), whilst it increases knowledge for participants and constitutes a cornerstone for support and capacity-building (VanWynsberghe et al., 2011). In other words, participation and informal exchange should be fostered as a means to encourage informal interaction among individual residents and generate social capital. This requires, as Chaskin (2001) notes, the development of engaged individuals *via* opportunities for participation, or the strengthening of associational networks *via* community organizing.

### A Transdisciplinary Theory of Morphosynthesis

**Figure 1:**
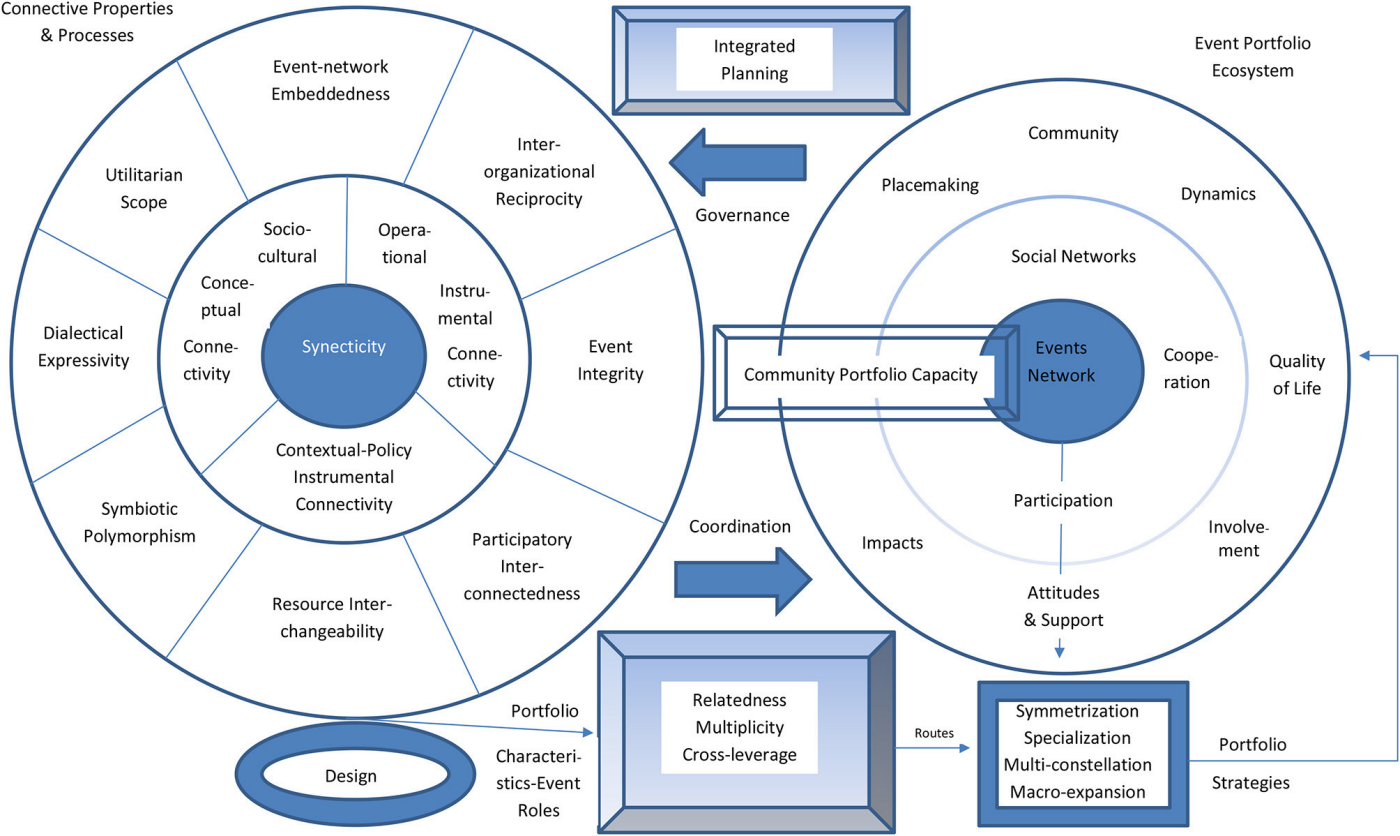
Morphosynthesis of event portfolio.

Etymologically, the term “morphosynthesis” derives from two Greek words that mean “shape” and “mixture” or “fusion,” respectively. As such, the term aims to capture the underlying processes of event portfolio co-creation, joint making, and cooperative production. This crafting aims to reconfigure an array of events (and their elements) into a new coherent whole that yields more value than the sum of its individual parts. Accordingly, portfolios can be understood as complex adaptive systems. Figure 1 depicts a schematic illustration of morphosynthesis conceptualized as a bidirectional interaction of two systemic spheres that connect the event portfolio with the substantive context of the host community, and thereby embed it into local networks, inter-organizational dynamics and placemaking policies. The interlinked spheres encompass the primary conditions, driving logics, and institutional environment lying beneath the establishment of structural arrangements that bring forth the primary management requirements of event portfolios.

In particular, the one sphere concerns the relationship of the community structures and networks with the portfolio that, in turn, determine capacity-building processes. This comprises the environment, infrastructure, actors and stakeholders that constitute the event portfolio ecosystem alongside the critical factors of local resources, market demand, competition, geographical location and seasonality, industry capability and community aptitudes (Ziakas, 2014a; Antchak et al., 2019). At the core of the ecosystem lies the events network, as this is the mechanism that engages social networks and organizations in portfolio planning and delivery. This may occur through inducing cooperation and encouraging participation in portfolio implementations. In so doing, necessary preconditions are the adoption of participatory planning and creation of a discursive public sphere enabling reflexive deliberation among actors and stakeholders. The outer part of the sphere describes the intersection of the whole community, its event-related dynamics and residents' portfolio involvement, its placemaking policy, the portfolio impacts on residents' quality of life and their subsequent attitudes and support for the portfolio. Of critical importance here is the establishment of multistakeholder governance structures and coordination schemes (like a consortium, forum, focal managing entity, etc.) in order to achieve the necessary integrated strategic planning for a portfolio. The other sphere concerns the means for galvanizing the portfolio character and pertinent qualities having at its core the process of synecticity (not to be confused with the technique of synectics); the concept refers to the connective properties and processes giving shape to the portfolio. These include the conceptual and instrumental connectivity that combines the socio-cultural, contextual, policy, and operational grounds of a portfolio. The makeup of synecticity involves a number of components essential for integrated portfolio planning, namely: event-network embeddedness, inter-organizational reciprocity, event integrity, participatory inter-connectedness, utilitarian scope, dialectical expressivity, symbiotic polymorphism, and resource inter-changeability.

The above components and properties of synecticity can be considered as the DNA of a portfolio, which enable coordination, and ultimately, contribute to its community capacity-building processes. They also substantially influence the design of the portfolio (i.e., reach, frequency, placement, size, and fit) and determine its relatedness, multiplicity, and efforts for cross-leverage. Internally, the composition of an event portfolio is shaped by the cultivation of design characteristics, which need to be regulated and balanced in order to achieve their coherent and harmonious functioning (Ziakas, 2014a; Antchak et al., 2019). As such, these characteristics can be thought of as in a continuum ranging across two antithetical value points:

- Formality of processes (from amorphous to standardized);
- Intentionality of actions (from purposive to unintended);
- Replicability of event elements (from mimetic to innovative);
- Anchored Polysemy of event meanings (from organic/endogenous to artificial/exogenous);
- Connectedness among events (from singular to multiplex);
- Directionality of portfolio approaches (from supply-side market-led to demand-side community engagement);
- Rhythmicity of adaptation (from intensive to passive).

Each event can play a different or synergistic role within a portfolio. The roles of events can be distinguished into functional and qualitative. Functional roles include events as core attractions, focal celebrations, complementary features, catalysts, and image-makers (Getz, 2005, 2012; Ziakas, 2014a, 2019b). Qualitative roles can be of iterative or pulsar nature (Antchak et al., 2019). Iterative events are periodic community-embedded events that bring attendees together regularly to bolster social relationships and produce bonding social capital (Richards, 2015). Pulsar events, on the other hand, are potential moments of change that can lead to the development of new structures, links, and opportunities (Richards, 2015). Pulsar events are large-scale international festivals and sporting tournaments that deliver dynamic changes in the host destination. A balanced combination of pulsar and iterative events in the portfolio provides a diverse spectrum of experience for both locals and visitors and develops eventfulness (Richards, 2015). Richards explains the interchange between iterative and pulsar events in Barcelona's portfolio, which connects the local spaces with the global flows succeeding to create a range of impacts such as image change, tourism growth, and urban regeneration. The combination of iterative and pulsar events within a portfolio can produce a change of pace and a diversity of experience bringing together various stakeholders and target audiences (Antchak et al., 2019).

Based on this common ground, the development of a portfolio can pursue one of the following strategies: symmetrization, specialization, multi-constellation, and macro-expansion (Ziakas, 2019b). Symmetrization is the proportional grouping of events by using a pyramid model to make a balanced portfolio of events that categorizes them in terms of type and scale. Thus, the portfolio composition is symmetrical consisting of a bulk of small-scale and fewer medium-scale events, and only some sporadic large-scale events. Opposite to symmetrization, the strategy of specialization focuses principally on distinct kinds of events and related ends that they can attend. For instance, there can be portfolios specializing in business, sport, cultural events or major events and economic, tourism, community or sport development, or ones seeking to reach niche markets. Multi-constellation is a strategy relating to a great diversity in portfolio composition by incorporating a largely heterogeneous and asymmetrical range of event types. Last, but not least, macro-expansion is a strategy intended to broaden the portfolio reach and size spreading its impacts and strategic outcomes to wider metropolitan or national areas. A variation of this strategy can be the creation of multiple portfolios in the same geographical area. Multiple portfolios can also facilitate collaboration among neighboring communities permitting them to leverage their own event portfolios conjointly and thereby spreading out their impacts to wider areas.

The effective implementation of portfolio strategies depends on cooperative exchanges within the events network, community participation in the portfolio as well as positive local attitudes and support. The ultimate purpose of these strategies is to enhance residents' quality of life, which in turn, would strengthen the role of the portfolio in the community, foster social networks, enhance support for the portfolio, and thereby, contribute to community portfolio capacity-building. In this multifactorial process, the event-related dynamics of power, public trust and participatory planning intermingle, influencing portfolio delivery and overall outcomes. Without a doubt, seeking the support of several community groups and encouraging the involvement of locals is not an easy task. Even when this happens, for example in the form of a local coalition, this may suffer from gaps in support and mistrust among other potential constituencies because the everyday, small-scale endeavors to build capacity are not prominent enough (VanWynsberghe et al., 2011). Still, the importance of actors' empowerment is pivotal for achieving community engagement and participation in events (Jepson et al., 2013), and for enhancing their trust to event-related entities (Ziakas and Costa, 2010a) through establishing interpersonal ties based on reciprocity and mutual trust (Mackellar and Jamieson, 2015).

The positive impact that quality of life may have on community social networks, portfolio capacity and participation suggests that it functions as an antecedent of community engagement and support for an event portfolio. This thesis concurs with the recent turn in tourism studies to consider quality of life as an antecedent of support for further development (Woo et al., 2015; Ouyang et al., 2019; Li et al., 2020). As such, quality of life can be construed as a 2-fold driver of resident attitudes. It does not only describe the existing standards of socio-economic conditions that characterize a community and determine attitudes or ability of residents to participate in event portfolio planning, but also it describes the expectation of residents that the portfolio will improve their quality of life. Hence, considering quality of life as an antecedent of community participation helps explain the dynamic interrelationships among attitudes, involvement, participation, and support as a part of capacity-building and social capital development.

## Concluding Thoughts

In this paper, I have brought together a range of theory fragments on event portfolio planning and management, integrated with the community capacity-building perspective, to formulate a transdisciplinary conceptual lens termed as morphosynthesis. This analytical and comprehensive approach theorizes the connective properties and processes that shape portfolios and enable their capacity to interlink attendant networks and the host community. Table 1 outlines the major concepts, alongside their definitions, that underpin the perspective of event portfolio morphosynthesis. This approach can ground further interdisciplinary inquiries on event portfolios, hence, eventually building a new autotelic subdiscipline cutting across event management, leisure, sport, culture, and tourism. To this end, an integrative ontogenetic groundwork has already been laid by Ziakas and Getz (2020, 2021) outlining the convergence of disciplines, theories and concepts alongside the foundational premises and subject realms of event portfolio management as a transdisciplinary field.

**Table 1:** Lexicon of relational synecticity and morphosynthesis of event portfolios.

**Concept**	**Definition**	**Synecticity units**	**Contextual designation**
Relatedness	The ways that events in a portfolio complement one another	Knowledge transfer, theming, volunteer pools, and target markets	Event portfolio definition
Multiplicity	Portfolio capacity to engender and convey multiple meanings and serve multiple purposes	Meanings and purposes	
Cross-leverage	The implementation of joint event strategies intended to achieve multiple outcomes	Strategies and tactics	
Conceptual connectivity	The sociocultural ground of an event portfolio encompassing and expressing different viewpoints	Meanings and symbolisms	Synergistic dynamics
Instrumental connectivity	The contextual operational and policy ground of an event portfolio in terms of sharing common elements, objectives, and resources	Common elements, objectives, and resources	
Event-network embeddedness	The overlap between social and economic linkages that influence event implementations and leveraging strategies as well as the nesting of event-related linkages within other social relationships	Common interests and kinship links	Organizational antecedents
Inter-organizational reciprocity	The deployment and common utilization of resources, facilities, skills, knowledge, and human labor in portfolio planning and delivery	Joint decision-making and problem-solving	
Event integrity	The consistency of portfolio events to express authentic community values and respond to participants' needs	Internal dimension of events having consistent cultural fit with the host community. External dimension of events consistently meeting the expectations of participants.	
Participatory inter-connectedness	Active and reflexive participation in portfolio activities resulting in esoteric development, (re)construction of shared meaning and fostering of relationships	Meaningful and deep social ties	
Utilitarian scope	The common sense of purpose that guides the formulation of event leveraging strategies and legitimizes them to the public discourse	Community needs and problems	Implementation parameters
Dialectical expressivity	The symbolic expression of worldviews and exchange of ideas through unrestricted dialogue	Metaphoric public discourse	
Symbiotic polymorphism	The synergetic multiformity of events in the portfolio that links them into a coherent whole	Event elements, themes, and meanings	
Resource inter-changeability	The capacity of using the same resources for different events within the portfolio	Resource interdependencies	
Events network	The non-institutionalized array of organizations that make decisions and take actions regarding planning and implementation of events in a host community as well as tend to engage in relationships that facilitate their goals	Trust, reciprocity, information exchange, resource-sharing, joint initiatives and joint problem-solving	Inter-organizational relationships
Symmetrization	The proportionate clustering of events, where a pyramid model is used to create a balanced portfolio by classifying events in terms of their type and scale	Symmetrical portfolio composition	Development strategies
Specialization	The focus on predominantly particular types of events and associated purposes that they can serve	Asymmetrical portfolio composition	
Multi-constellation	The portfolio composition exhibiting high variety by encompassing a broadly varied and asymmetrical array of event types	Asymmetrical portfolio composition	
Macro-expansion	The spatial expansion and magnitude dispersion of a portfolio to wider metropolitan or national areas as well as the management of multiple portfolios in a geographical area or through collaboration among adjacent communities	Symmetrical or asymmetrical portfolio composition	
Formality	The extent to which standardized operating procedures, written rules and policies, and official documentation records of events' activities are put in place	From amorphous to standardized	Portfolio characteristics
Intentionality	The extent to which all the procedures, activities, and communicative scope in the portfolio are strategic and intended to achieve certain objectives	From unintentional-spontaneous to purposeful-deliberate	
Replicability	The propensity to replicate entire events or certain event elements in a portfolio	From mimetic adoption to innovative creation	
Anchored-polysemy	The extent to which the variety of symbolic meanings extracted from an event portfolio are organically innate or are being constructed and imposed by exogenous actors	From endogenous-organic to exogenous-artificial	
Connectedness	The ways by which events in the portfolio are connected to one another	From singular to multiplex	
Directionality	The orientation of portfolio design approaches, either on the supply-side market-led initiatives or demand-side community engagement in portfolio design	From market-led to local-led	
Rhythmicity	The ability of city event managers to modify their design portfolio approach due to the contextual changes and revision of objectives	From intensive to passive	
Pulsar events	Large-scale one-off events that can lead to the development of new structures, links, and opportunities	Change and bridging of social networks	Qualitative event roles
Iterative events	Periodic community-embedded events that can maintain community networks by bringing people together on a regular basis to cement strong social ties	Stability and bonding of social networks	
Core attractions	Events as anchors for attracting tourist visitation	Tourist visitation	Functional event roles
Focal celebrations	Events as anchors for achieving community development	Community consolidation	
Complementary features	Events as supplementary attributes that add combined value to the portfolio	Harmonizing effects	
Image-makers	Events as a means for destination brand building	Brand associations	
Catalysts for development	Events as facilitators for development and placemaking	Stimulant enabling development functions	

The perspective of morphosynthesis is an antidote to the incessant over-fragmentation of the event industry and has practical merit as it explores heuristic means for cultivating and harnessing the social value of event portfolios. Accordingly, it highlights the critical importance of community participation in fostering social capital, as also indicated elsewhere (Schulenkorf, 2012; Mackellar and Jamieson, 2015). If portfolio managers employ inclusive participatory planning involving and empowering locals in decision-making, they can develop the public sphere as a space of dialogue, participation and exchange, thereby building relationships based on trust and cooperation, increase residents' engagement in further participation, enhance their support for events, and finally, contribute to community capacity-building. Since speedy action to deliver events successfully, prevents debate and engagement (Dredge and Whitford, 2011), it is critical to identify the most efficient means and conditions that can foster dialogue and residents' participation in event portfolio planning. From this perspective, it is equally significant to consider how quality of life influences residents' support and capacity-building for portfolios.

In conclusion, morphosynthesis is a distinctive event-based theory elaborating on the integrative portfolio nature. It has a relational focus amalgamating the contextual, policy, operational, and sociocultural grounds of portfolios. In essence, morphosynthesis reveals the bidirectional relationship between portfolios and social order pointing out how portfolios are being shaped by the extant social networks of the host community, and in turn, how portfolios influence the community's social order, building local capacity and prompting social action. Through this lens, the interaction of connective portfolio properties is viewed as a whole, incorporating complex dynamic and adaptive patterns that shape them as social systems. At the core of this multifactorial and multilevel process lie the synecticity and the supporting events network that enable the interconnection of social networks and the community through the array of events. Future empirical work can shed light on the particularities and circumstances that influence and moderate this underlying functioning of portfolios and their subsequent contribution to creating social value for host communities.

## Author Contributions

The author confirms being the sole contributor of this work and has approved it for publication.

## Conflict of Interest

VZ was employed by company Leisure Insights Consultancy Ltd. The author declares that the research was conducted in the absence of any commercial or financial relationships that could be construed as a potential conflict of interest.

## Publisher's Note

All claims expressed in this article are solely those of the authors and do not necessarily represent those of their affiliated organizations, or those of the publisher, the editors and the reviewers. Any product that may be evaluated in this article, or claim that may be made by its manufacturer, is not guaranteed or endorsed by the publisher.
